# Implementation of Provider-Based Electronic Medical Records and Improvement of the Quality of Data in a Large HIV Program in Sub-Saharan Africa

**DOI:** 10.1371/journal.pone.0051631

**Published:** 2012-12-17

**Authors:** Barbara Castelnuovo, Agnes Kiragga, Victor Afayo, Malisa Ncube, Richard Orama, Stephen Magero, Peter Okwi, Yukari C. Manabe, Andrew Kambugu

**Affiliations:** 1 Infectious Diseases Institute, Makerere College of Health Sciences, Kampala, Uganda; 2 Division of Infectious Diseases, Department of Medicine, Johns Hopkins University School of Medicine, Baltimore, Maryland, United States of America; University of Ottawa, Canada

## Abstract

**Introduction:**

Starting in June 2010 the Infectious Diseases Institute (IDI) clinic (a large urban HIV out-patient facility) switched to provider-based Electronic Medical Records (EMR) from paper EMR entered in the database by data-entry clerks. Standardized clinics forms were eliminated but providers still fill free text clinical notes in physical patients’ files. The objective of this study was to compare the rate of errors in the database before and after the introduction of the provider-based EMR.

**Methods and Findings:**

Data in the database pre and post provider-based EMR was compared with the information in the patients’ files and classified as correct, incorrect, and missing. We calculated the proportion of incorrect, missing and total error for key variables (toxicities, opportunistic infections, reasons for treatment change and interruption). Proportions of total errors were compared using chi-square test. A survey of the users of the EMR was also conducted. We compared data from 2,382 visits (from 100 individuals) of a retrospective validation conducted in 2007 with 34,957 visits (from 10,920 individuals) of a prospective validation conducted in April–August 2011. The total proportion of errors decreased from 66.5% in 2007 to 2.1% in 2011 for opportunistic infections, from 51.9% to 3.5% for ART toxicity, from 82.8% to 12.5% for reasons for ART interruption and from 94.1% to 0.9% for reasons for ART switch (all *P*<0.0001). The survey showed that 83% of the providers agreed that provider-based EMR led to improvement of clinical care, 80% reported improved access to patients’ records, and 80% appreciated the automation of providers’ tasks.

**Conclusions:**

The introduction of provider-based EMR improved the quality of data collected with a significant reduction in missing and incorrect information. The majority of providers and clients expressed satisfaction with the new system. We recommend the use of provider-based EMR in large HIV programs in Sub-Saharan Africa.

## Introduction

Real-time, provider-based electronic medical records (EMR) have been used extensively for health care in developed countries. They have been shown to improve legibility of clinical notes [1], patient safety [2] and quality of care [3]. They have also been introduced in Sub- Saharan Africa [4] and been successful in reducing patient waiting time and in increasing the time spent with the providers [5]. Provider-based EMR could be a valuable aid to health care providers working in HIV programs in resource-limited settings for clinic management and reporting, especially in urban clinics where large numbers of patients are registered [4]. For example touch screen aids have been used for voluntary counseling and testing, and have been shown to increase completeness and accuracy of data. In addition, they eliminate the need for retrospective entry which saves time and resources. Data is also available for monitoring and evaluation of clinic programs [6]. Therefore, provider-based EMR may be an attractive solution for HIV programs in Sub-Saharan Africa [4], [7] especially in busy, urban clinics.

The Infectious Diseases Institute (IDI) is an urban clinic in Kampala, Uganda [8], [9] with more than 27,000 adult patients cumulatively registered by December 2011, of which over 12,000 have been started on ART. Data collected in the clinic database is used for clinical care, internal audits, generation of reports for partners and stakeholders, as well as planning for drug procurement. Despite local ethics committee approval in 2009 to use IDI routine clinical data for analysis and publications, such data has been shown to have high rates of errors and missing information [10], [11], [12], [13], [14] and, therefore, may not be suitable for research purposes.

**Figure 1:**
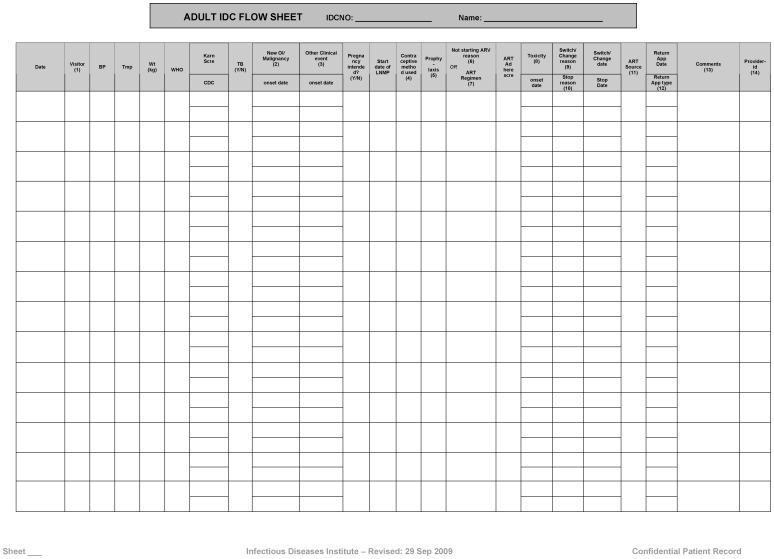
Standardized medical forms. Forms completed by health care providers and subsequently entered in an electronic database before the implementation of provider-based electronic medical records.

Prior to 2009, the IDI used both paper-based free text and standardized forms (Figure 1) completed by health care providers and subsequently entered in an electronic tool by data clerks to populate a patient database. This approach led to high rates of missing and inaccurate data in both steps, which were 1) the providers transcribing form the notes correct and 2) complete quantitative information onto the standardized forms and the data clerks entering this quantitative information in the database. [11]. In 2010 to present, the IDI introduced phased solutions leading to a provider-based EMR that was complimented with a structured process of data validation.

From November 2010 until July, 2011, all data collected in the HIV clinic was entered into both the standardized forms and the provider-based EMR to enable validation of the entered data. In March 2011, data entry by providers was enhanced, standardized forms were eliminated but doctors and nurses continued filling free text clinical notes (where any relevant finding is highlighted), which are used to validate the information in the database by two quality-control nurses.

To our knowledge, there is no data available from Sub-Saharan Africa showing the effect of provider-based EMR on the correctness and completeness of the data collected. The main objective of this study was to compare the rate of errors in the IDI routine HIV care database before and after the introduction of provider-based EMR. We also assessed the acceptability of provider-based EMR by the users and clients through a qualitative questionnaire. Finally we propose a transition model from paper based records filled by providers to real time provider-based EMR.

## Methods

### Development of Provider-based EMR

Prior to the development of the current IDI EMR, systems in the region and worldwide were considered but rejected due to inability to customize to IDI clinic requirements (e.g. not open-source, rigid workflow, unable to handle large volumes of data). In September 2007, the IDI implemented a custom-made EMR system called Integrated Clinic Enterprise Application (ICEA). Provider-based entry was an important planned feature to reduce the rate of errors, provide real time validation of data, and automate tasks such as drug prescription writing. The following principles were considered when developing ICEA: efficiency of clinical workflow, continuity of care, quality of care and information, confidentiality, information security and storage, as well as flexibility for additional applications such as new subspecialty clinic data.

### System Architecture

ICEA is a *Microsoft® (MS)Windows* forms application that is based on *Microsoft.NET* technologies and developed in C# with a MS SQL Server backend. The application was developed by a team of software developers based at the IDI and it is not open source. Steps of the development of the application (including the source code) are well documented from envisioning, conceptual design, logical design and physical design to enable anyone who understands these standards (including *Windows* coding language) and has access to the documentation to maintain and extend the application. The server computer is a HPDL380 G7 series and the client computers are as well HP PCs.

To enable provider-based entry, ICEA had to be visually compelling and user-friendly. Workflow management was in-built to enable tracking of patients from the time their visit was registered to visit triage, counseling session, medical examination, drug prescription and collection. To eliminate omission of important steps, such as scheduling the patient next visit, automated queries were created that were mandatory. Many fields are mandatory and must be filled-in before the record can be considered valid and saved. Moreover, there are internal consistency checks which ensure that the data entered is accurate and falls within required ranges while maintaining the integrity of the information. Each user in the system has to explicitly login on a computer and into ICEA which is joined to the IDI domain using their username and password. Each user falls within a user group and each group is enabled to specific tasks only (e.g. medical officers are not able to access the inventory of pharmacy stock). Information collected in ICEA database server is backed up daily onto a secondary server and weekly backed up onto tapes which are stored outside the IDI building.

### Features

The system has a modular design which enables plug-ins to be developed and installed without affecting the existing system. This feature has enabled the phased development of additional modules to meet additional clinical needs (for example a module to house the integrated tuberculosis clinic database was one such addition).

**Figure 2:**
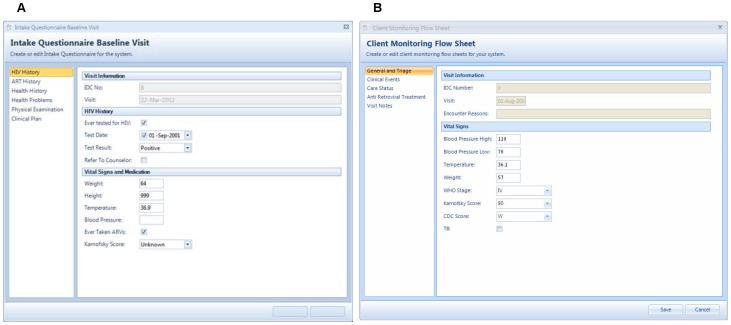
Intake questionnaire (A) and client monitoring flow sheet (B). Provider-based electronic medical records as they appear in the Integrated Clinic Enterprise Application.

The clinical patient management application is the main feature of ICEA. Modules built in this application are described in Table 1 and shown in Figure 2.

**Table 1:** Description of Integrated Clinic Enterprise Application modules for clinical patient management.

Module	Function
Patient clinic registration	To register patients in the clinic and collect information of past medical history
Patients inactivation	To inactivates patients transferred out, lost to follow up, or deceased
Visit registration and triage	To register patient visit and record vital signs and presenting symptoms
Monitoring	To enter and access the information collected during the follow up visits including: Karnosky and WHO staging, new clinical events and opportunistic infections, last menstrual period, contraceptives methods, prophylaxis, ART regimen, reasons for switching and stopping ART, ART toxicities, ART adherence
Appointment scheduling	To schedule appointments and prevent overbooking
Lab	To order laboratory tests
Counseling	To collect socio-economic, marital, religious and sexual behavior data in a rule based approach
Prescription	To prescribe and dispense drugs

WHO: World Health Organization; ART: antiretroviral treatment.

Other features are:

1. A client overview that summarizes relevant clinical information such as ART regimen, opportunistic infections, allergies and CD4 count results displayed in both a table and graph form (Figure 3).

**Figure 3:**
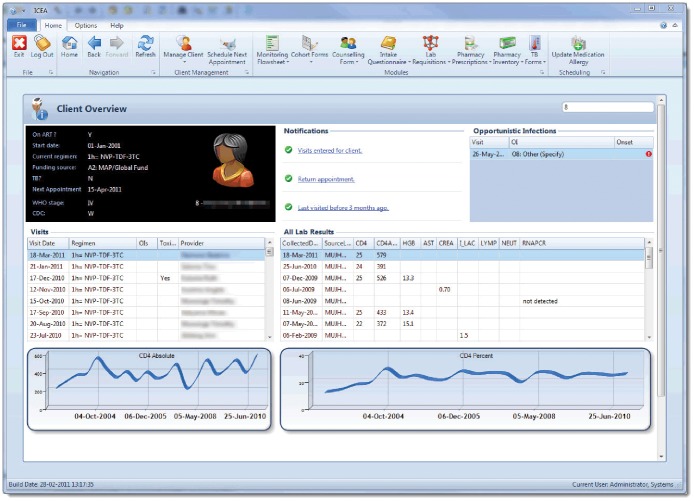
The patient overview. Summary of patient relevant clinical information as they appear in the Integrated Clinic Enterprise Application.
2. Laboratory results are downloaded automatically into the ICEA database from the laboratory (running on a separate system) using an MS SQL Integration Services package.
3. A specialized provider-based EMR for patients co-infected with tuberculosis
4. A specialized provider-based EMR for patients with Kaposi’s sarcoma (under development)
5. A specialized provider-based EMR for pregnant women (under development)
6. Inventory management and management of drugs
7. Integrated bug reporting and auto upgrading on new versions.
8. Finally ICEA has a reporting server based on SQL Server Reporting Services which has consistently grown to have more than 200 reports to enable efficient management of the clinic and reporting to the Ministry of Health, the Centers for Disease Control and other stakeholders.

### Assessment of Quality of Data

We assessed four key variables for errors prior to the new ICEA provider-based EMR and after its implementation: opportunistic infections, reason for antiretroviral treatment (ART) regimen discontinuation, reason for ART change (e.g. one or more drugs are substituted on that visit without ART interruption), and ART toxicities. Codes for the 4 key variables did not substantially change before and after the implementation. Errors were classified as missing information or incorrect information. We calculated a total error rate by dividing the combined number of missing and incorrect events by the total number of events that could have been coded for that field in randomly selected patient charts or over the pre-determined time interval.

The error rates before provider-based EMR (2007) were obtained by a retrospective comparison of the information on both the paper-based clinical notes and the standardized forms of 100 randomly selected patients on ART with the information entered in the clinic database [11]. After the provider-based EMR implementation, the error rates were obtained through a prospective validation from April to August 2011 by comparing the information in the clinic notes (considered as “relative gold standard” [15]) with the information entered in the clinic database. Training and guidelines were available to clinicians on what events should be clearly stated in the free text, for example if an opportunistic infection had occurred and entered into the provider-based EMR, it should be clearly stated in the notes.

Extractions for both studies were performed by experienced nurses and trained medical students to minimize errors in the process.

We used Chi square tests to test for differences between proportions of errors for the different variables. A *P-*value <0.05 was considered significant. Statistical analysis was carried out using STATA 11.2 (Texas, USA).

### Qualitative Assessment

We assessed the acceptability of the provider-based EMR through two separate anonymous questionnaires which were administered to both providers and clients. Providers and clients selected for the survey were individuals working or attending the clinic before and after the introduction of the real time provider-based entry EMR. The answers were categorized as follows: 1) favorable, if the respondent had chosen the options: “I strongly agree” and “I agree”; 2) neutral, if the respondent had chosen “Neither agree nor disagree”; and 3) unfavorable if the respondent had chosen the option “Disagree’ or “Strongly Disagree’. Summary statistics of information collected were generated.

This study was approved and annually renewed by the School of Medicine Research and Ethics Committee, Makerere University Medical School (#2009-120), and the Uganda National Council for Science and Technology. According to the approved protocol participants are not consented since this is routinely collected data used for clinical care; no personal identifiers are seen by the researchers.

## Results

### Deployment of Real Time Provider-based Entry EMR

By August 2010, computers were connected to uninterruptible power supplies (UPS) and installed in each of the clinician rooms and connected to the local area network; 25 providers were trained on the use of ICEA. Telephone extensions were installed in each of the clinician rooms. This enabled efficient communication with the system developers. The cost (in US$) of the in-house development and deployment of the system was $3,647 for the software, $45,235 for the hardware, $18,935 for consultancy, $50,012 for permanent staff time for a total of 117,829 US$.

### Comparison of Quality of Data Before and After Real Time Provider-based EMC

We compared the results from 2,382 visits from 100 individuals of the 2007 audit with 34,957 visits from 10,920 individuals of the 2011 audit (from April to August). Table 2 shows the comparison of the two validation exercises. The total error decreased from 66.5% in 2007 to 2.1% in 2011 for opportunistic infections, from 51.9% to 3.5% for ART toxicity, from 82.8% to 12.5% for reasons for ART interruption and from 94.1% to 0.9% for reasons for ART switch (all *P*<0.0001).

**Table 2:** Comparison of the proportion of errors in the Infectious Diseases Institute Clinic database before and after the implementation of the Integrated Clinic Enterprise Application.

Variable	Number Missing (%)		Number Incorrect (%)		Total Error (%)		P*
	2007	2011	2007	2011	2007	2011	
New opportunistic infections	124/227 (54.6)	9/469 (1.9)	27/103 (26.2)	1/460 (2.1)	151/227 (66.5)	10/469 (2.1)	<0.0001
ART toxicity	220/453 (48.6)	8/226 (3.5)	15/223 (6.7)	0/218 (0)	235/453 (51.9)	8/226 (3.5)	<0.0001
Reasons for ART interruption	18/35 (51.4)	1/16 (6.2)	11/17 (64.7)	1/15 (6.7)	29/35 (82.8)	2/16 (12.5)	<0.0001
Reasons for ART switch	23/51 (45.1)	2/411 (0.4)	7/28 (25)	2/409 (0.4)	48/51 (94.1)	4/411 (0.9)	<0.0001

* P values were calculated for the difference in total error.

ART: antiretroviral treatment.

### Surveys

**Figure 4:**
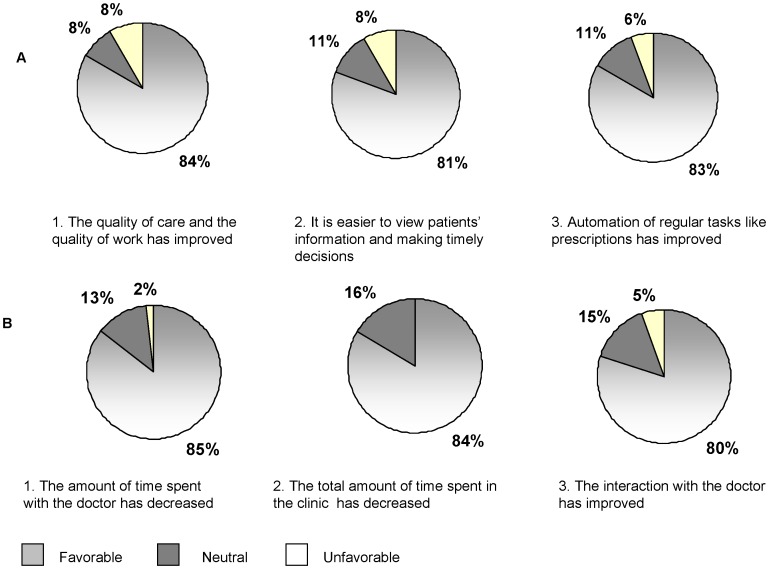
Survey on acceptability. Answers to 3 significant questions by providers (A) and clients (B).

The questionnaires were administered in August 2011. Thirty-six providers (7 doctors, 10 nurses, 7 counselors, 4 laboratory technicians, and 7 pharmacy staff, 1 did not identified him/herself) were randomly chosen among the 80 eligible for the survey; 55 clients out of 8,434 eligible were selected through convenience sampling during a period of 5 clinic working days. Sixty-nine percent of the providers gave a favorable answer when asked if, in general, the clinical services had improved since the start of real-time provider-based entry EMR. Figure 4a shows the results from 3 key questions in the survey; specifically 30 (84%) gave a favorable answer regarding the quality of care and work; 81% gave a favorable answer regarding accessing patient information and making timely decisions, and 83% regarding automation of regular tasks such as booking appointments, requesting lab tests and filling pharmacy prescriptions. All clients agreed or strongly agreed that the real time provider-based EMR contributed positively to clinic services; the majority gave favorable answers regarding the doctor-patient interaction and the reduction of time spent in the clinic and with the providers (Figure 4b).

## Discussion

In the last decade electronic medical records have become widely available in resource-limited settings [5], [16], [17], [18]. In Sub-Saharan Africa, urban HIV programs are often congested, resulting in long waiting times [8], [9] and information storage constraints; provider-based EMR is an attractive solution to some of these challenges [4], [17].

This is the first study conducted in Sub-Saharan Africa to investigate the accuracy of data collected with user-based EMR in an HIV program. In our study we found that with the user-based EMR the rates of errors range from 0.9 to 12.5% depending on the variable. In addition the introduction of user-based EMR led to a reduction in the total error rates from 66.5% to 2.1%. Before the implementation of user-based EMR, the majority of errors for three of the four variables investigated were due to incompleteness, and particularly to information collected in the clinical notes but not entered by the providers in the standardized form, and consequently not made available to data clerks for entry. This suggests that skipping the data entry step and making providers the only responsible of data quality by the implementation of a user-based EMR can be greatly minimize missing information. Although mandatory fields have the danger of information being invented or faked to be able to continue with data entry, such instances, which would be labeled as “incorrect” information during the consistency checks carried out through prospective validation occurred at a low rate. Improved quality of data and the utilization of a user-based EMR could also translate into improvement in patient care, provider efficiency, human resource savings, and improved accuracy of reports [19].

However other factors should be taken in consideration to explain this drastic decrease in errors rates. Although the staff force did not substantially changed, as well as national guidelines for ART treatment in adults (except for phasing out stavudine in 2008 and introducing more slots of tenofovir), given the long period between the evaluations, there is no doubt that the clinic staff gained knowledge and experience, as well as data quality awareness. In 2008, with the aim of improving the quality of data, a real time prospective quality assurance process was introduced and despite improvement, the rate of errors was still unacceptable leading to a huge amount of workload to rectify errors.

Overall, the provider-based EMR by the clinic staff was rated positively, with 4 in 5 providers agreeing that the implementation of provider-based EMR resulted into improvement of the quality of care, access to patients’ information in order to make timely decisions, and automation of regular tasks such as prescription writing. Of note, while data entry staff was phased out, providers did not receive any additional incentive to enter the information collected during patients’ visits; therefore their level of satisfaction genuinely reflects improved working conditions. The addition of intelligent checks into system (e.g. the use of alerts to guide prescription writing), the automation of some tasks, and the availability of reports that summarize patient care and treatment history are some of the features most appreciated by providers. It should be emphasized that the developers of ICEA involved health care providers from the planning stage through the deployment of the system. In addition, they continue to offer ongoing technical assistance and dialog with the providers and can make modifications and additions to the systems if clinically required.

Although our patient population is largely computer-illiterate and could have misperceived this innovation, our patients welcomed provider-based EMR and they perceived that it improved the quality of care and reduced the time spent with the providers filling forms. This was confirmed by the comparison of two time motion surveys conducted in our program that showed a decreased doctor and nurse visit time after the implementation of the provider-based EMR.

Overall the ICEA system had several important strengths. First, the system was built in- house by system developers familiar with the local setting, HIV clinical care, the IDI clinic, patient management and workflow. Furthermore, it also resulted into cost savings. Second, the system guarantees completeness of the EMR, not only because all fields must be filled before saving a record, but also because different modules have to be saved in a rigid order; for example, information in the monitoring module cannot be entered if the registration module is not filled, or an electronic prescription cannot be filled if there is no monitoring information and a next appointment is not scheduled.

There are other advantages of ICEA and provider-based EMR not investigated in this study. The first one is the increased legibility since the information can be accessed as a text rather than handwritten clinical notes. The second is record keeping: paper records could be completely eliminated, with the advantage of eliminating the need for storage space and records clerk personal. Information is also always available to the providers through the database.

**Figure 5:**
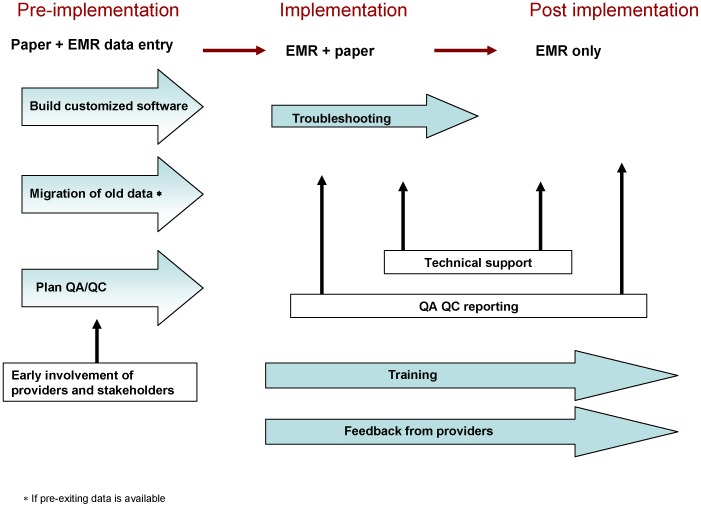
Transition from paper forms to provider-based EMR in our program.

In Figure 5, we diagram the transition from paper forms to provider-based EMR in our program. Although a direct comparison can no longer be made because of changeover of most of the providers, in the first quarter of 2012 the rate of errors for all the four variables evaluated in this study were less than 1%. Given the very low error rate, the program may consider embracing a record system based exclusively on provider-based EMR.

Not surprisingly and similar to other programs in similar settings [5], [20], the implementation of ICEA faced some barriers to change. Among providers other than doctors, computer literacy was low and some providers felt intimidated by computerization. Each group of providers was then separately trained accordingly to the level of knowledge they needed to perform their task. After the formal training, a team of tutors was available to assist users on real operational work.

Secondly, even when computer knowledge was not a barrier, some providers were initially concerned that that information stored in a server could not be as secure as information stored in hard copies [4]. This misconception was overcome when they were taught about backup systems and also given a password. Providers also realized that even if the patient file containing the clinical notes was misplaced, patient management was still possible through the EMR. In our experience, the phase-in period when both paper and user-based EMR were filled in is necessary for the providers to gain confidence in the new system despite retarding work flow. However it is still possible that reluctance in abandoning completely the paper-based clinical notes constitutes a barrier to implementation of exclusively provider-based EMR.

A thorough user analysis and early involvement of the users ensured that the product reflected their needs and likely reduced the risk of provider rejection or poor compliance when it was implemented**.** Finally, a strong management [21], and support from system developers and information technology specialists are key components for a satisfactory transition from paper to user-based EMR.

In conclusion, the introduction of provider-based EMR had a favorable impact on the quality of data collected with a significant reduction in missing and incorrect information. The improvement of quality of the routinely collected data has the potential to impact care and lead to more accurate data for research purposes. The majority of both providers and clients expressed satisfaction with the new system. We recommend the use of provider-based EMR in large, urban HIV programs in Sub-Saharan Africa.
